# Comparative machine learning models for predicting the compressive strength of ultra-high-performance concrete

**DOI:** 10.1371/journal.pone.0357153

**Published:** 2026-08-28

**Authors:** Nguyen Thi Thu Nga, Nguyen Anh Tuan, Nguyen Tuan Tu, Huyen Thi Dang

**Affiliations:** 1 Advanced Materials and Smart Structure for Sustainable System (AMS4), University of Transport Technology, Hanoi, Vietnam; 2 Faculty of Civil Engineering, University of Transport Technology, Hanoi, Vietnam; 3 School of Information & Communications Technology, Hanoi University of Industry, Hanoi, Vietnam; 4 UCT Company, University of Transport and Communication, Hanoi, Vietnam; King Mongkut’s University of Technology North Bangkok, THAILAND

## Abstract

Ultra-high-performance concrete (UHPC) exhibits exceptional mechanical properties and durability. However, its compressive strength is highly dependent on complex mix design parameters. While traditional experimental techniques and regression-based models are commonly used to evaluate UHPC compressive strength, machine learning approaches offer an efficient alternative for capturing complex nonlinear relationships. This study develops a machine learning–based framework to predict the compressive strength of UHPC and compares the predictive performance of five advanced algorithms: Extremely Randomized Trees (ER), Light Gradient Boosting Machine (LightGBM), Extreme Gradient Boosting (XGBoost), CatBoost, and Artificial Neural Network (ANN). A comprehensive experimental database was utilized for training and validation purposes. Among the evaluated models, CatBoost achieved the best predictive performance, with a coefficient of determination (R²) exceeding 0.90, a root mean square error (RMSE) of approximately 4.5 MPa, and a mean absolute error (MAE) of approximately 3.6 MPa. However, subgroup residual analysis showed that the prediction reliability was not uniform across the full strength range. In particular, mixtures with compressive strength ≥180 MPa exhibited larger errors and systematic underprediction, mainly due to the limited number of ultra-high-strength samples in the compiled database. Therefore, the model is more reliable within well-represented strength ranges, while predictions in the ultra-high-strength region should be interpreted with caution. SHAP-based analysis, feature dependency analysis, and both Individual Conditional Expectation (ICE) and Partial Dependence Plots (PDP) were employed. These explainable AI techniques identified key variables and quantified their contributions to the compressive strength of UHPC. The findings demonstrate that interpretable machine learning can support preliminary UHPC mixture assessment by combining predictive performance with physically meaningful insights.

## 1. Introduction

Ultra-high-performance concrete (UHPC) is a new generation of cementitious composite materials [1]. UHPC exhibits exceptional compressive strength, ductility, and durability compared with conventional concrete. Optimizing granular packing density, maintaining a low water-to-binder ratio, and incorporating additional cementitious ingredients, including fly ash, slag, metakaolin, silica fume, and steel or synthetic fibers, are key factors contributing to its exceptional performance [2]. UHPC has been increasingly applied in high-performance structural elements, bridge components, and architectural applications. UHPC enables long-term performance and reduced maintenance.

Despite its excellent mechanical performance, the design of UHPC mixtures remains challenging. The compressive strength of UHPC is governed by complex interactions among water content, cementitious materials, silica fume, mineral additions, aggregate gradation, superplasticizer dosage, fiber parameters, specimen size, and testing-related conditions. Conventional empirical or regression-based approaches are often insufficient to capture these nonlinear and coupled effects [3]. In addition, experimental optimization of UHPC mixtures is usually time-consuming, material-intensive, and costly. Therefore, reliable data-driven approaches are useful for reducing experimental workload and supporting mixture design [4].

In recent years, machine learning (ML) techniques have emerged as powerful tools in civil and materials engineering [5–9]. It enables accurate prediction of concrete properties and uncovers hidden patterns in experimental datasets [10]. Recent reviews have shown that ML models can effectively capture nonlinear relationships among material composition, processing parameters, and mechanical performance in composite materials [11,12]. In concrete and cementitious composites, ML-based models have been used to estimate compressive, tensile, and flexural strengths, elastic modulus, fracture-related properties, and durability indicators [13,14]. UHPC poses additional modeling challenges due to its multi-component binder system, ultra-low porosity, and high sensitivity to mixture proportions [15]. This requires more advanced algorithms capable of capturing subtle nonlinearities and feature interactions [16]. Alaneme et al. [17] looked into the application of Artificial Neural Networks (ANN), Adaptive Neuro-Fuzzy Inference Systems (ANFIS), and Gene Expression Programming (GEP) to predict the compressive and flexural strengths of geopolymer concrete containing sugarcane bagasse ash (SCBA) and banana peel ash (BPA). ANFIS outperformed other models. In a similar vein, Okasha et al. [18] estimated the elastic modulus and flexural strength of concrete containing carbon nanotubes (CNTs) using machine learning algorithms such as ANN, Support Vector Regression (SVR), and Histogram-based Gradient Boosting (HGB). They discovered that ANN produced the best modulus predictions. HGB was also better in terms of flexural strength. Using 85 data samples and 12 input variables pertaining to mix design and fiber characteristics, Rezvan et al. [19] created an ANN model to forecast the compressive strength of plastic fiber-reinforced concrete (PFRC) made from recycled PET bottles.

However, several limitations remain in existing ML-based studies on UHPC compressive-strength prediction. First, many studies focus mainly on improving aggregate prediction accuracy, while the reliability of models across different compressive-strength ranges is less frequently examined. This issue is particularly important for UHPC because ultra-high-strength mixtures are often under-represented in literature-compiled datasets. As a result, a model may show high overall accuracy while still producing relatively large errors in the high-strength region. Second, although multi-source experimental databases are commonly used, the effects of data heterogeneity, testing-related variables, specimen dimensions, and differences among original studies are not always sufficiently discussed. Third, critical limitation of most prior ML-based studies lies in the lack of model interpretability. Many high-performing ML algorithms, especially ensemble and deep learning models, are often treated as “black boxes.” They provide accurate predictions but limited physical insights into how each input parameter influences the output. For engineering design, interpretability is as important as accuracy. The integration of explainable artificial intelligence (XAI) techniques offers a promising pathway to bridge this gap between predictive modeling and materials science understanding.

In this study, a multi-source experimental database containing 28-day UHPC compressive-strength results was compiled and preprocessed. Five representative ML models, including Extremely Randomized Trees, XGBoost, LightGBM, CatBoost, and Artificial Neural Networks, were trained and evaluated under a consistent validation and hyperparameter-optimization procedure. Model performance was assessed using R², RMSE, and MAE. In addition to aggregate performance metrics, the predictive reliability of the best-performing model was further examined across different strength ranges to identify possible limitations in under-represented high-strength regions. XAI techniques, including SHAP-based feature importance, SHAP waterfall analysis, PDP, and ICE plots, were then used to interpret the influence of important input variables on UHPC compressive strength.

Although XGBoost, ANN, LightGBM, and other machine learning models have been previously used for predicting the compressive strength of UHPC and related cementitious composites, the added value of the present study lies in the integrated structure of the proposed framework rather than in the use of a single new algorithm. Specifically, this study combines multi-source UHPC data harmonization, consistent model benchmarking, strength-range-based reliability assessment, and physically interpretable XAI analysis within the same workflow. The methodological innovation is threefold. First, the experimental database was compiled from multiple published UHPC studies and processed using clearly defined mixture descriptors and testing-related covariates to improve consistency among different data sources. Second, all selected models were trained, tuned, and evaluated under the same preprocessing, cross-validation, and hyperparameter-optimization strategy, allowing a fair comparison of predictive performance. Third, model reliability was not assessed only through aggregate metrics, but was also examined across different compressive-strength ranges to identify possible limitations in under-represented ultra-high-strength mixtures. In addition, the XAI analysis was used not merely to rank input variables, but to connect model behavior with UHPC material mechanisms and mixture-design interpretation. Therefore, the proposed framework provides a transparent and engineering-oriented approach for UHPC compressive-strength prediction, supporting both numerical accuracy and data-informed understanding of mixture-design factors.

Accordingly, this study aims to compile and preprocess a multi-source database of 28-day UHPC compressive strength with clearly defined mixture- and testing-related variables. It then benchmarks representative tree-based, gradient-boosting, and neural-network models under a consistent preprocessing, hyperparameter-optimization, and validation framework. Beyond evaluating aggregate predictive performance, the study examines model reliability across different compressive-strength ranges, with particular attention to the under-represented ultra-high-strength region. The most influential variables governing UHPC compressive strength are subsequently identified, and the learned model behavior is interpreted using SHAP, PDP, and ICE analyses. Through this integrated approach, the study seeks to combine predictive accuracy with physically meaningful interpretation to support transparent and data-informed UHPC mixture assessment.

## 2. Methodological background

### 2.1. Database and Pre-processing

A comprehensive experimental database was compiled to develop and validate the machine learning models for predicting the compressive strength of UHPC. The database was from published experimental studies on UHPC mixtures, with compressive strength data available at 28 days. After a systematic review of the literature [1,2,20–38], the dataset was collected.

To ensure the reliability and consistency of the multi-source database, the data collection and screening procedure was conducted in three main steps. First, experimental data were collected from published UHPC studies that reported mixture proportions and 28-day compressive strength. Only studies providing sufficient quantitative information on binder composition, water content, supplementary cementitious materials, aggregate characteristics, chemical admixtures, fiber-related parameters, specimen size, and compressive strength were considered. Data reported only in graphical form or without clear mixture-design information were not included unless the numerical values could be reliably identified from the original source.

Second, the collected records were screened to remove duplicate mixtures, incomplete entries, inconsistent units, and physically unrealistic values. All mixture components were converted into comparable units before model development. Records with missing essential variables or unclear compressive-strength values were excluded rather than estimated, in order to avoid introducing artificial uncertainty into the database.

Third, the cleaned database was validated through statistical and engineering-consistency checks before model training. Descriptive statistics, distribution plots, and correlation analysis were used to identify abnormal values, extreme imbalance, and possible redundant variables. The retained data were also checked against typical UHPC mixture-design ranges based on engineering judgment. Values that were inconsistent with physically meaningful UHPC proportions or testing conditions were removed. Specimen size was retained as a testing-related covariate because the database was compiled from different experimental studies using different specimen geometries. However, it was interpreted as a control variable rather than an intrinsic mixture-design parameter.

After this screening and validation process, the final database contained 379 UHPC mixtures with 16 input variables and one target variable, namely the 28-day compressive strength. Although this procedure improves the consistency of the compiled dataset, the database still reflects the inherent heterogeneity of literature-reported experimental data. Therefore, the potential influence of multi-source variability was considered when interpreting model performance and the reliability of predictions in different strength ranges. Table 1 shows the data collection and screening process.

**Table 1:** Data collection, screening, and validation procedure for the UHPC database.

Step	Purpose	Procedure applied in this study
Data collection	To obtain relevant UHPC mixtures from published studies	Experimental studies reporting mixture proportions and 28-day compressive strength were selected. Only records with sufficient quantitative information on mixture composition, specimen size, and strength were retained.
Unit harmonization	To ensure comparability among different sources	Mixture components were converted into consistent units. Powder materials, water, and superplasticizer were normalized with respect to the total cementitious material content.
Record screening	To remove unreliable data	Duplicate mixtures, incomplete records, unclear strength values, inconsistent units, and physically unrealistic values were excluded.
Variable definition	To reduce ambiguity in input features	The term “cm” was defined as total cementitious material mass. Specimen size was treated as a testing-related covariate rather than a UHPC material descriptor.
Data validation	To check data reliability before modeling	Descriptive statistics, distribution plots, correlation analysis, and engineering-consistency checks were used to identify abnormal values and confirm that the retained records were physically plausible.

Some important UHPC-related variables, including curing temperature, curing regime, specimen shape, testing standard, fiber type, and fiber tensile strength, were not consistently reported in the source studies. Therefore, these variables could not be included as independent input features without substantially reducing the database size or introducing uncertain assumptions. Their absence is acknowledged as a limitation and may contribute to residual prediction errors. To partially represent testing- and fiber-related effects, specimen size, steel fiber volume, and fiber aspect ratio were retained as input variables. Since only 28-day compressive-strength data were used, curing age was controlled by data selection rather than modeled as an input feature.

Other testing-related factors, such as curing temperature, curing regime, specimen shape, loading rate, and compressive-strength testing standard, were not consistently reported across all original studies. Therefore, these factors could not be introduced as independent input variables without substantially reducing the database size or introducing uncertain assumptions. Instead, their possible influence was treated as part of the inherent heterogeneity of the multi-source experimental database. This limitation was considered when interpreting model accuracy, particularly for underrepresented strength ranges.

The input parameters include cement, cement type, fly ash, slag, silica fume, metakaolin, nano-silica, limestone powder, quartz powder, sand, maximum aggregate size, water, superplasticizer dosage, steel fiber volume, fiber aspect ratio, and specimen size. To avoid ambiguity in variable definitions, all input variables were explicitly defined before model development. In this study, the abbreviation “CM” denotes the total cementitious/binder material mass used as the reference denominator, not the mass of cement alone. Therefore, variables such as cement, fly ash, slag, silica fume, metakaolin, nano-silica, limestone powder, quartz powder, water, and superplasticizer are expressed as ratios relative to the total CM content. The variable “cement type” represents the reported strength grade of cement used in the original experimental studies, expressed in MPa where available. It was included as a material-related descriptor to reflect differences in cement grade among the collected UHPC mixtures. Steel fiber volume was expressed as the volume fraction of steel fibers, while the fiber aspect ratio was defined as the ratio between fiber length and fiber diameter. Specimen size represents the characteristic dimension of the compressive-strength test specimen and was included as a testing-related covariate rather than an intrinsic mixture-design parameter. Table 2 presents the definition, physical meaning, and unit or normalized form of each variable used in the UHPC compressive-strength prediction models.

**Table 2:** Definition of input variables used in the UHPC database.

Variable	Symbol	Definition	Unit/ form
Cement	C	Mass of cement divided by total CM content	Cement-to-CM ratio
Cement type	CT	Reported cement strength grade	MPa
Fly ash	FA	Mass of fly ash divided by total CM content	Fly ash-to-CM ratio
Slag	SL	Mass of slag divided by total CM content	Slag-to-CM ratio
Silica fume	SF	Mass of silica fume divided by total CM content	Silica fume-to-CM ratio
Metakaolin	MK	Mass of metakaolin divided by total CM content	Metakaolin-to-CM ratio
Nano-silica	NS	Mass of nano-silica divided by total CM content	Nano-silica-to-CM ratio
Limestone powder	LP	Mass of limestone powder divided by total CM content	Limestone powder-to-CM ratio
Quartz powder	QP	Mass of quartz powder divided by total CM content	Quartz powder-to-CM ratio
Sand	S	Mass of sand divided by total CM content	Sand-to-CM ratio
Maximum aggregate size	MAS	Maximum particle size of aggregate	mm
Water	W	Mass of water divided by total CM content	Water-to-CM ratio
Superplasticizer	SP	Mass of superplasticizer divided by total CM content	Superplasticizer-to-CM ratio
Steel fiber volume	SFV	Volume fraction of steel fibers in the mixture	%
Fiber aspect ratio	FAR	Ratio of fiber length to fiber diameter	Dimensionless
Specimen size	SS	Characteristic dimension of the compression test specimen	mm
Compressive strength	CS	28-day compressive strength of UHPC	MPa

Table 3 presents the statistical characteristics (minimum, maximum, mean, and standard deviation) of these parameters after data cleaning.

**Table 3:** Descriptive statistics of the UHPC dataset.

Variable	Count	Mean	Std. Dev.	Min	Median	Max	Skewness	Kurtosis
C	379.0	0.75	0.14	0.17	0.79	1.0	−1.46	2.49
CT	379.0	48.84	4.82	42.5	52.5	52.5	−0.56	−1.7
FA	379.0	0.05	0.11	0.0	0.0	0.5	2.25	4.22
SL	379.0	0.03	0.09	0.0	0.0	0.7	3.66	14.35
SF	379.0	0.16	0.08	0.0	0.18	0.33	−0.59	−0.67
MK	379.0	0.01	0.04	0.0	0.0	0.29	4.48	20.72
NS	379.0	0.0	0.01	0.0	0.0	0.17	6.31	59.95
LP	379.0	0.02	0.09	0.0	0.0	1.09	6.67	57.67
QP	379.0	0.08	0.13	0.0	0.0	0.79	1.59	2.73
S	379.0	1.01	0.42	0.0	1.0	2.07	−0.31	0.68
MAS	379.0	2.23	1.78	0.08	2.0	5.0	0.61	−1.16
W	379.0	0.19	0.03	0.1	0.19	0.29	0.45	1.66
SP	379.0	0.03	0.02	0.0	0.02	0.24	6.37	63.0
SFV	379.0	1.05	1.44	0.0	0.0	6.56	1.58	2.85
FAR	379.0	37.79	53.52	0.0	37.5	400.0	4.03	25.35
SS	379.0	48.94	17.04	40.0	40.0	110.0	2.25	3.97
CS	379.0	139.07	32.93	57.01	141.47	217.0	−0.14	−0.12

**Fig 1:**
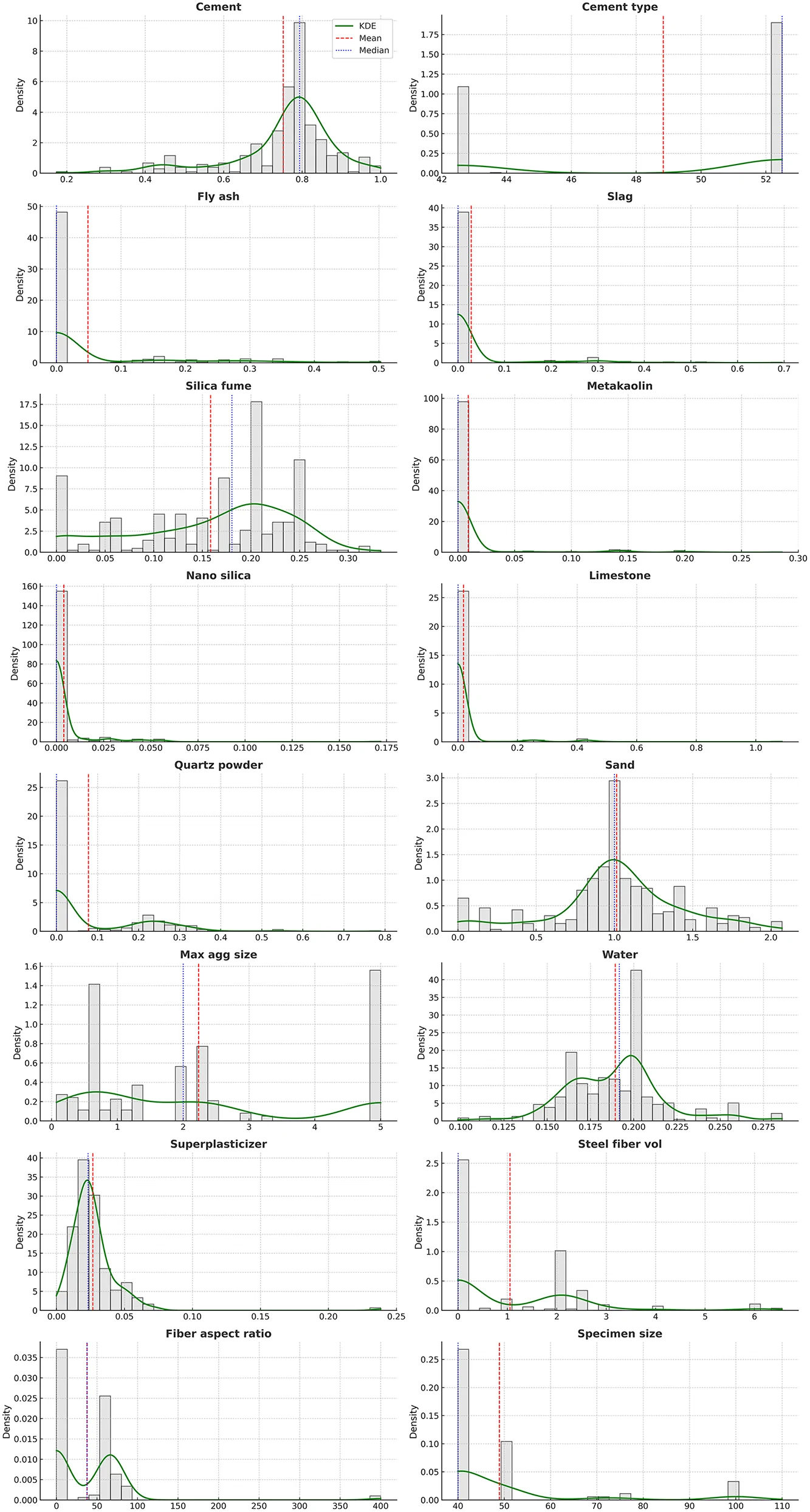
Statistical distributions of cementitious materials and supplementary components in the UHPC database, including cementitious materials, supplementary powder components, water, superplasticizer, aggregate-related variables, fiber-related variables, specimen size, and compressive strength.

The compressive strength of UHPC in the database ranges from 57.01 MPa to 217.0 MPa, with an average value of 139.07 MPa. This reveals a significant variation in mix composition and mechanical performance across different studies. The variability provides a robust basis for model training and generalization. The independent variables also exhibited diverse statistical distributions, reflecting different mix design philosophies and raw material sources. Distribution and correlation analysis were performed to ensure the quality of the data. The statistical distributions of the input variables are illustrated in Fig 1.

The histograms combined with kernel density estimation (KDE) curves provide insights into the variability and balance of the dataset. Most of the input variables exhibit right-skewed distributions, reflecting the practical ranges typically used in UHPC mix designs. Exploratory plots confirmed that the dataset was physically plausible, providing a solid foundation for developing powerful machine learning models.

**Fig 2:**
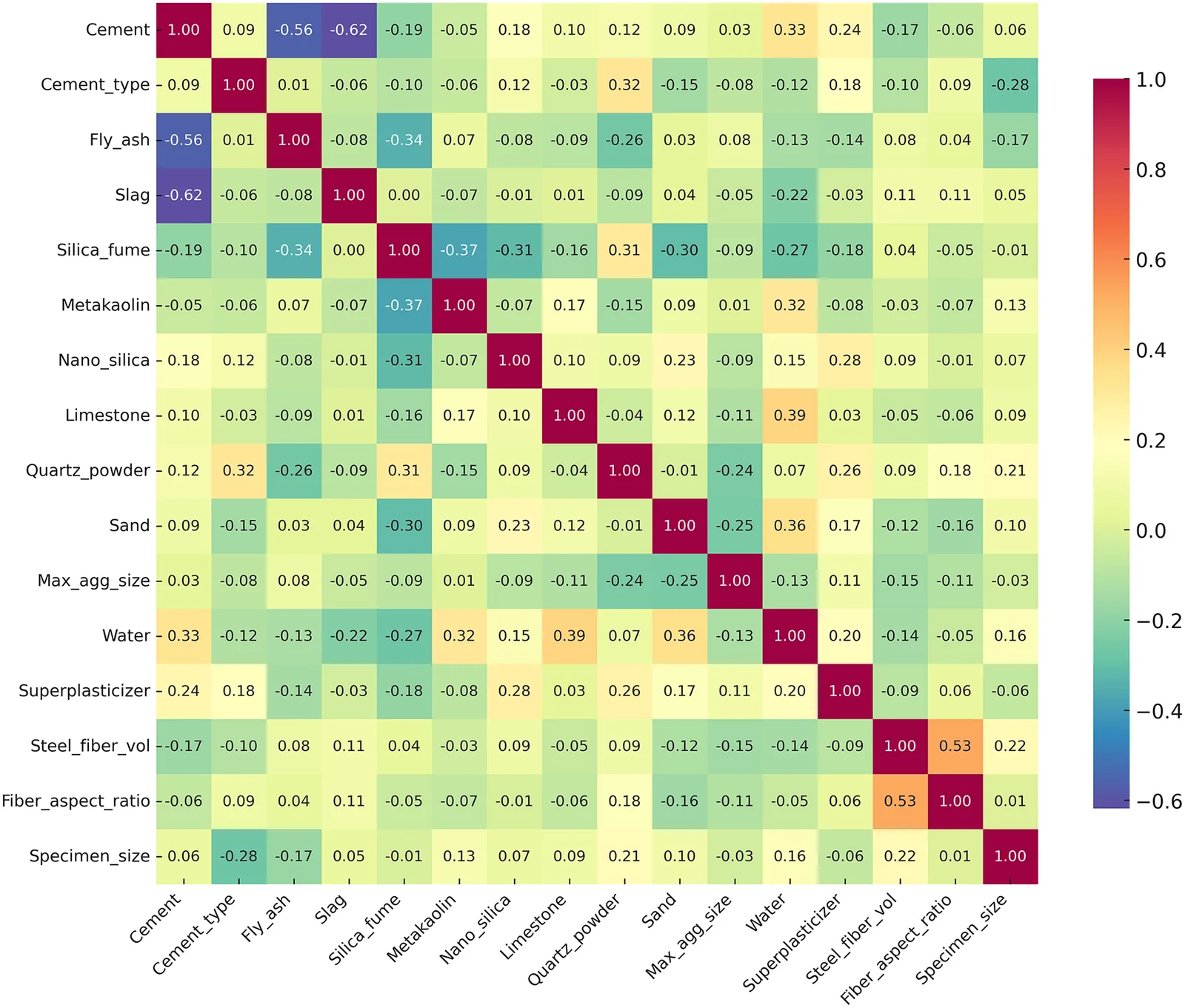
Pearson correlation heatmap among input variables in the UHPC dataset.

The relationship between the input variables is tested using the Pearson correlation coefficient, illustrated in Fig 2. The findings indicate that no dominant collinearity exists among the predictors, ensuring a stable input space for subsequent model training and reliable feature importance analysis.

Data preprocessing steps were applied to improve data quality and ensure reliable model performance. The raw database, collected from various literature sources, contained variations in measurement units, incomplete records, and potential inconsistencies. The preprocessing procedure was implemented as follows: (1) Numerical features were normalized using min–max scaling, transforming each feature into a range between 0 and 1; (2) Categorical variables, where applicable, were transformed using Label Encoding; (3) The cleaned and normalized dataset was randomly divided into two subsets: 80% for model training and 20% for testing.

The final preprocessed dataset provides a balanced and comprehensive representation of the UHPC component. It allows for the comparative evaluation of multiple machine learning algorithms for compressive-strength prediction. The data preprocessing and distribution procedures ensure that the models can learn meaningful correlations and generalize well.

### 2.2. Methodology

The methodological framework of this study is designed to develop, train, and evaluate multiple machine learning (ML) algorithms for predicting the compressive strength of UHPC. The overall workflow consists of four main stages: (i) data collection and preprocessing, (ii) model selection and implementation, (iii) model evaluation and validation, and (iv) explainable artificial intelligence (XAI)-based interpretation.

#### 2.2.1. Model Selection and Implementation.

Five advanced algorithms were selected based on their proven effectiveness in handling nonlinear and high-dimensional data:

(1) Extremely Randomized Trees (ER): An ensemble-based model similar to Random Forests. ER constructs multiple decision trees by introducing greater randomness in both feature selection and threshold determination for splits. This reduces variance and improves model generalization while maintaining computational efficiency.
(2) LightGBM: A gradient-boosting framework that uses histogram-based decision tree learning. LightGBM is optimized for high-speed training and scalability on large datasets. Its leaf-wise growth strategy allows it to capture complex nonlinear relationships effectively.
(3) XGBoost: A widely used ensemble model that builds additive trees sequentially to minimize a specified loss function. XGBoost incorporates regularization terms and advanced optimization techniques, making it robust against overfitting and well-suited for tabular data engineering.
(4) CatBoost: A gradient-boosting algorithm using ordered target statistics and permutation-driven encoding. It automatically mitigates overfitting and requires minimal parameter tuning, resulting in superior performance on heterogeneous datasets.
(5) ANN: A multi-layer perceptron architecture was employed to model complex nonlinear interactions between input features and the compressive strength output. The network was trained using the backpropagation algorithm with the rectified linear unit (ReLU) activation function and the Adam optimizer. Hyperparameters, including the number of hidden layers, neurons, and learning rate, were optimized through a grid search and cross-validation process.

All models were implemented using Python and the Scikit-learn, LightGBM, CatBoost, and TensorFlow/Keras libraries. Hyperparameter tuning was performed to optimize model performance, utilizing randomized or grid search strategies in conjunction with 10-fold cross-validation.

Although the final database contains 379 UHPC mixtures, which is a moderate dataset size for machine learning applications in materials engineering, its adequacy depends on the complexity of the selected model. For tree-based ensemble and gradient-boosting models, the dataset size is considered acceptable because these models are generally effective for structured tabular datasets and can handle nonlinear feature interactions with relatively limited sample sizes. However, for ANN, the dataset is not large enough to justify a deep or highly complex architecture. Therefore, the ANN model was intentionally designed as a constrained feed-forward network rather than a deep neural network.

To reduce the risk of overfitting, several measures were adopted. The input dimensionality was kept moderate, with 16 physically meaningful mixture and testing-related variables. After that, the ANN architecture was limited to a small number of hidden layers and neurons, and its hyperparameters were selected through cross-validation rather than manual tuning. Early stopping and a maximum epoch limit were applied to prevent excessive fitting to the training data. For tree-based models, hyperparameter tuning was also used to control model complexity, including the number of estimators, tree depth, learning rate, and related regularization parameters.

The performance of the ANN was therefore interpreted within the scope of this constrained architecture and the available dataset size. The results indicate that, given the current dataset size and tuning strategy, boosting-based models were more effective and stable than the tested ANN configuration. A larger, more standardized UHPC database would be required to fully exploit more complex ANN or deep learning architectures and to further improve model generalization.

#### 2.2.2. Model evaluation and performance metrics.

The performance of the trained models was assessed using both quantitative and graphical evaluation methods. The following statistical indicators were used to evaluate predictive accuracy and generalization ability:

Coefficient of Determination (R²): Indicates how well the predicted values explain the observed variability in compressive strength.


$$\begin{array}{c} R^{\text{2}}=1-\frac{\sum {\left(y_{i}-{\hat{y}}_{i}\right)}^{\text{2}}}{\sum {\left(y_{i}-\overset{―}{y}\right)}^{\text{2}}} \end{array}$$ (1)

Root Mean Square Error (RMSE): Measures the magnitude of prediction errors, penalizing larger deviations more heavily.


$$\begin{array}{c} RMSE=\sqrt{\frac{\text{1}}{n}\sum {\left(y_{i}-{\hat{y}}_{i}\right)}^{\text{2}}} \end{array}$$ (2)

Mean Absolute Error (MAE): Represents the average absolute difference between predicted and observed values.


$$\begin{array}{c} MAE=\frac{\text{1}}{n}\sum \left|y_{i}-{\hat{y}}_{i}\right| \end{array}$$ (3)

In Eqs. (1) – (3), $y_{i}$ and ${\hat{y}}_{i}$ denote the experimentally measured and model-predicted UHPC compressive strengths for the i^th^ sample, respectively, $\overset{―}{y}$ represents the mean of the experimental values. The total number of samples is denoted by *n.* The coefficient of determination (R^2^) evaluates the proportion of variance in the experimental compressive strength that is explained by the predictive model. The root mean square error (RMSE) quantifies the square-root of the averaged squared differences between predicted and measured strengths. The mean absolute error (MAE) represents the average magnitude of prediction errors without emphasizing outliers.

In this study, R², RMSE, and MAE were used together because no single metric can fully characterize predictive performance. R² evaluates the overall goodness of fit, RMSE penalizes large prediction errors, and MAE reflects the typical absolute prediction error in MPa. Therefore, the combined use of these three indicators enables a balanced assessment of model accuracy, error severity, and practical prediction reliability.

#### 2.2.3. Explainable AI and Model Interpretation.

To better understand the physical significance of the predictive models and identify the most influential parameters affecting compressive strength, several explainable AI (XAI) techniques were applied, including:

- SHapley Additive exPlanations (SHAP): quantifying the contribution of each input feature to individual predictions
- Feature Dependency Analysis: exploring nonlinear relationships between features and target value
- Individual Conditional Expectation (ICE) and Partial Dependence Plots (PDP): visualizing how changes in single or multiple features affect the model output

## 3. Model implementation and evaluation

### 3.1. Model training and test procedure

**Fig 3:**
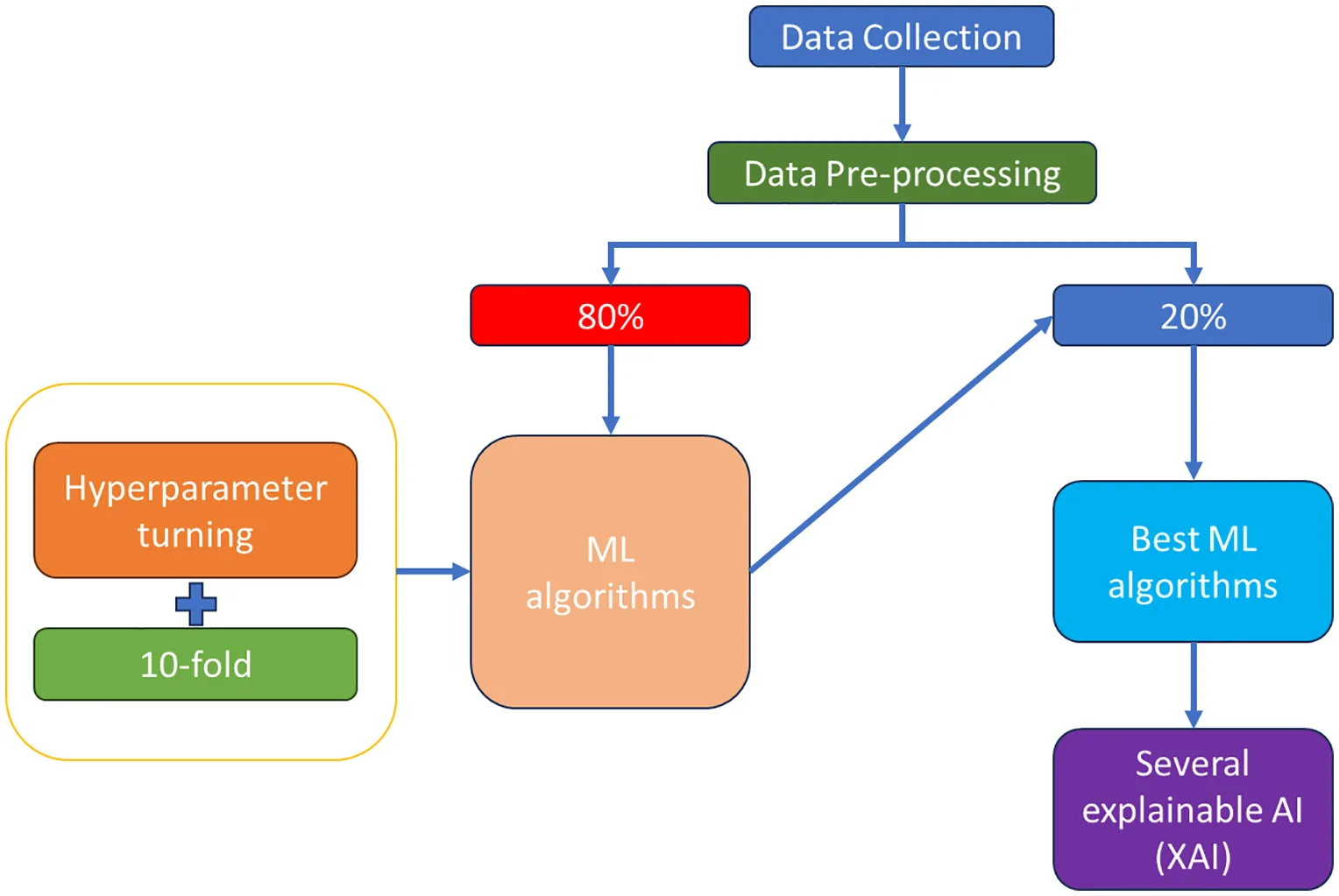
Model training and test procedure.

Fig 3 presents the workflow for constructing machine learning models that aim to predict compressive strength of UHPC. During model development, hyperparameter optimization was performed using Randomized Search for tree-based and boosting models and Grid Search for the ANN, combined with 10-fold cross-validation. The optimized hyperparameters are summarized in Table 4.

**Table 4:** Hyperparameter optimization settings and optimal configurations of the machine learning models.

Model	Optimization method	Hyperparameter	Search space	Optimal value	Selection criterion
Extremely Randomized Trees	Randomized Search + 10-fold CV	n_estimators	100–800	500	Lowest mean CV-RMSE
		max_depth	None, 5–30	None	
		min_samples_split	2–10	2	
		min_samples_leaf	1–5	1	
		max_features	sqrt, log2, 0.5–1.0	1.0	
XGBoost	Randomized Search + 10-fold CV	n_estimators	200–800	300	Lowest mean CV-RMSE
		max_depth	3–10	5	
		learning_rate	0.01–0.10	0.05	
		subsample	0.6–1.0	0.8	
		colsample_bytree	0.6–1.0	0.8	
LightGBM	Randomized Search + 10-fold CV	n_estimators	200–800	400	Lowest mean CV-RMSE
		num_leaves	16–64	31	
		learning_rate	0.01–0.10	0.05	
		max_depth	−1, 3–10	−1	
		subsample	0.6–1.0	0.8	
CatBoost	Randomized Search + 10-fold CV	iterations	200–800	500	Lowest mean CV-RMSE
		depth	4–8	6	
		learning_rate	0.01–0.10	0.05	
		l2_leaf_reg	1–10	3	
ANN	Grid Search + 10-fold CV	Hidden layers	1–3	2	Lowest mean CV-RMSE
		Neurons per layer	32–128	64, 32	
		Activation function	ReLU, Tanh	ReLU	
		Batch size	16–64	32	
		Learning rate	0.0001–0.01	0.001	
		Epochs	100–300	200	

Hyperparameter optimization was performed to ensure a fair comparison among the selected machine learning models and to reduce the risk of overfitting [39]. All models were tuned using a 10-fold cross-validation strategy on the training dataset. The testing subset was not used during hyperparameter optimization and was reserved only for final model evaluation. The optimal hyperparameter configuration was selected based on the lowest mean cross-validated RMSE, because RMSE directly reflects the magnitude of prediction errors in MPa and penalizes large deviations more strongly than MAE.

Randomized Search was adopted for the tree-based ensemble and boosting models, including Extremely Randomized Trees, XGBoost, LightGBM, and CatBoost, because these models have relatively broad hyperparameter spaces and Randomized Search allows efficient exploration with reduced computational cost. Grid Search was used for the ANN model because the number of predefined architectural parameters was more limited. The search spaces were defined based on commonly used ranges in previous machine learning applications for composite and cementitious materials, as well as preliminary trial runs. The final search spaces and selected optimal values are summarized in Table 4.

To fully evaluate predicted accuracy and reliability, performance is quantified using a variety of statistical metrics, including as R^2^, RMSE, MAE. The contribution of each input variable to the predicted compressive strength is then ascertained by analyzing the algorithm exhibiting the best performance using SHAP values.

### 3.2. Evaluation of Model

**Fig 4:**
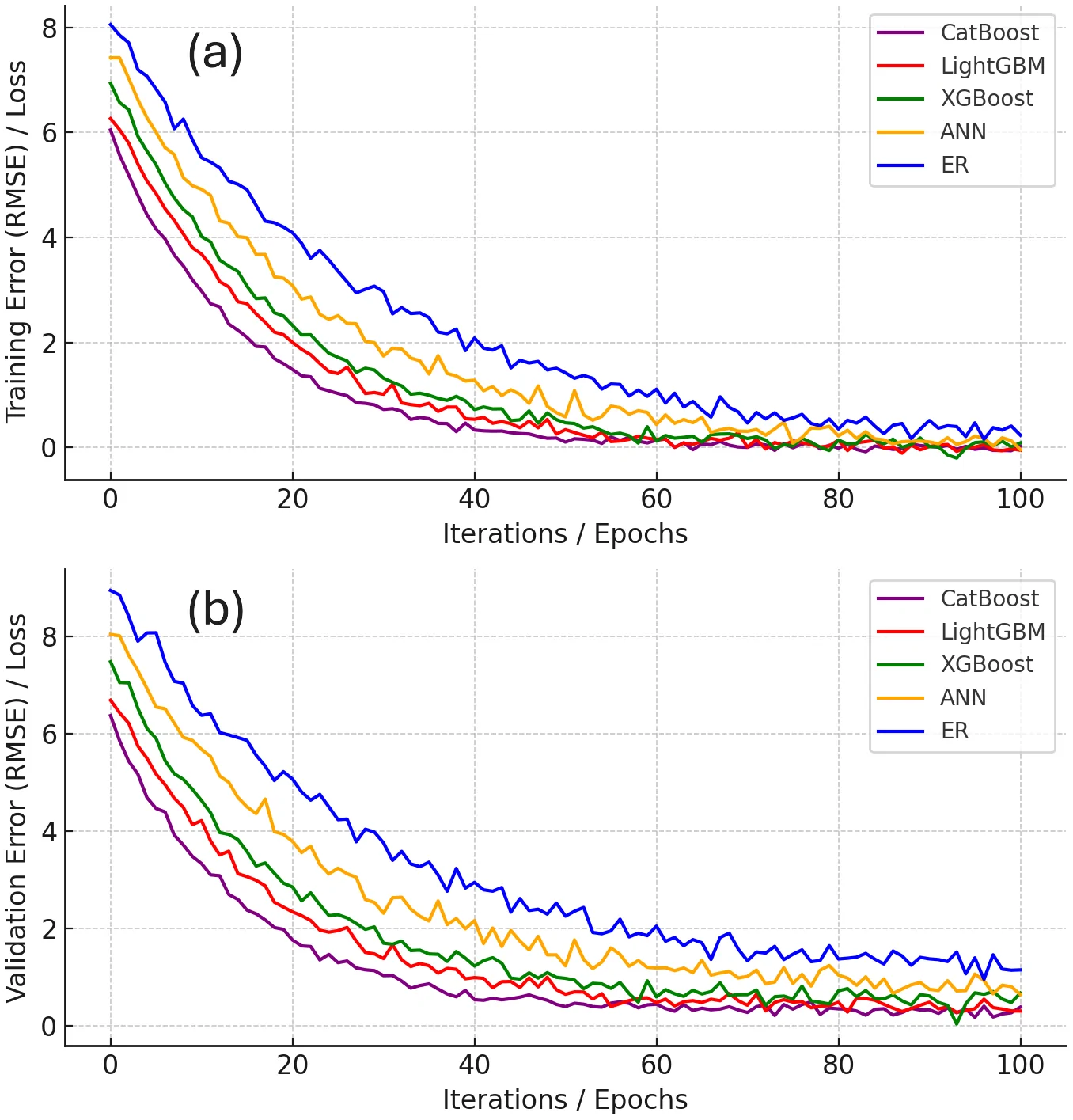
Learning curve of machine learning models: (a) training; (b) Validating.

Fig 4 illustrates the learning curves of the five ML models during both the training and validation processes.

The results clearly show a general decreasing trend in the error (RMSE/Loss) as the number of iterations increases. All models converge and learn the basic relationship between UHPC mix design parameters and compressive strength. During the training phase (Fig 4a), all models show a rapid decrease in error in the initial 20–40 iterations. Among the tested models, CatBoost achieves the lowest training error and the fastest convergence rate. LightGBM and XGBoost follow closely behind. The ANN model shows slower convergence and higher residual error. The ER model consistently records the highest training error, reflecting its limited ability to minimize bias compared to boosting-based methods. During the validation phase (Fig 4b), similar convergence patterns were observed. CatBoost still performs best

**Fig 5:**
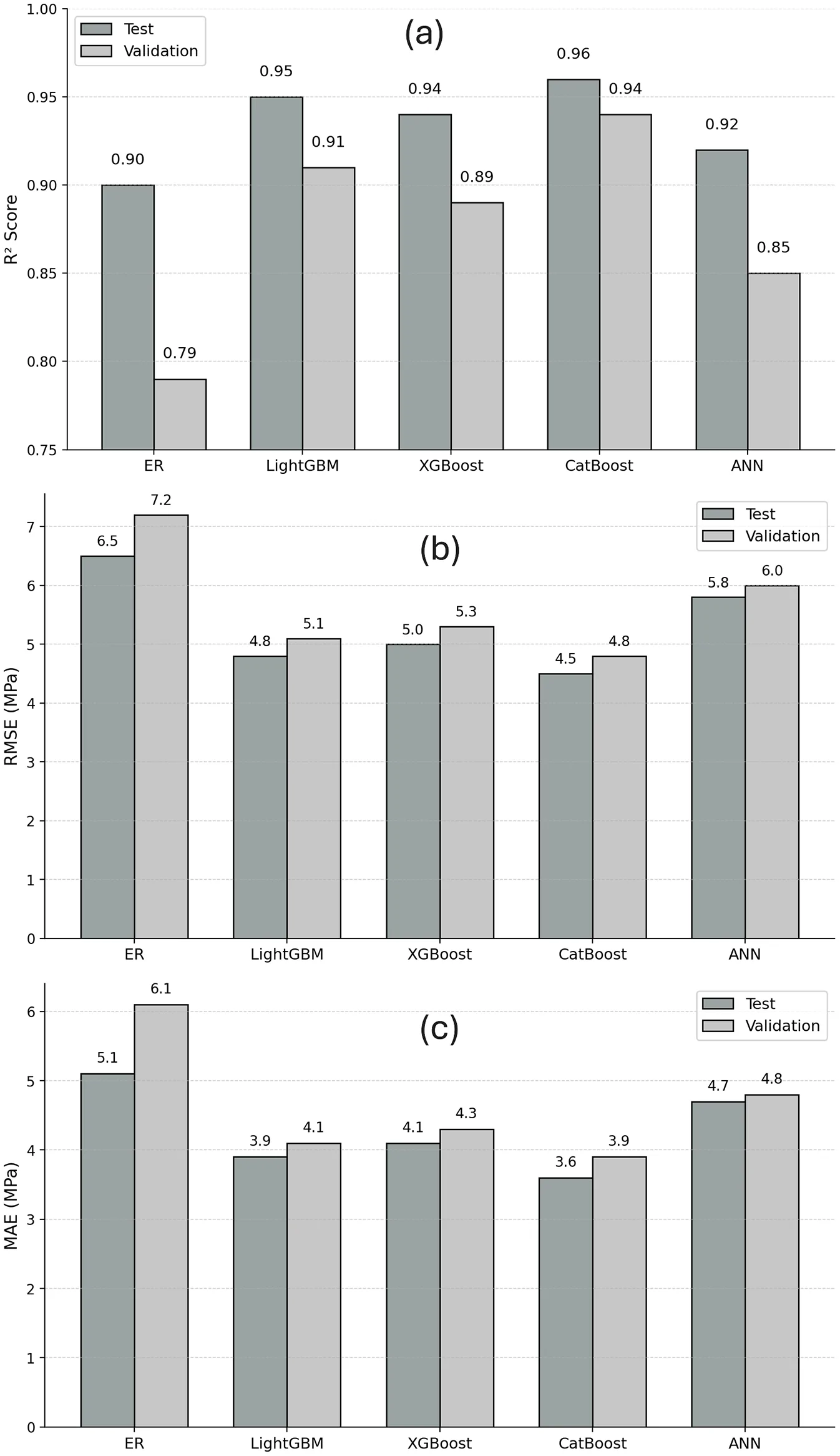
Comparison of model performance based on (a) coefficient of determination (R²); (b) RMSE and (c) MAE.

In Fig 5a, the test R² values indicate generally good predictive performance, with CatBoost achieving the highest score. However, the validation R² values were lower for ER and ANN, indicating weaker generalization compared with the boosting-based models.

The error-based comparison in Fig 5b further reinforces these findings. With an RMSE of 4.5 MPa and an MAE of 3.6 MPa, the CatBoost model produced the least amount of error. With RMSE values less than 5.0 MPa, the LightGBM and XGBoost models likewise produced comparatively low errors. ANN and ER, on the other hand, showed greater biases, with ER having the greatest RMSE (6.5 MPa) and MAE (5.1 MPa). This implies that the intricate multivariate interactions present in UHPC mixtures are more difficult for ER to capture.

In comparison with existing machine learning studies on UHPC compressive strength, which commonly report R² values in the range of approximately 0.85–0.93 and RMSE values exceeding 5 MPa for heterogeneous datasets, the predictive performance achieved in this study is competitive. It should be noted that direct numerical comparisons are influenced by differences in dataset composition, input variables, and modeling strategies.

Although the CatBoost model shows strong aggregate performance, its predictive reliability is not uniform across the full UHPC strength spectrum. To examine the effect of target-distribution imbalance in this literature-compiled dataset (379 samples) and to avoid over-interpreting aggregate metrics, we performed a class-wise residual analysis using repeated stratified hold-out validation, with strength classes defined as <100 MPa, 100–150 MPa, 150–180 MPa, and ≥180 MPa. The dataset is strongly concentrated in the 100–150 MPa range (203/379 samples, 53.6%), whereas only 40 samples (10.6%) belong to the ≥ 180 MPa region. This imbalance substantially limits the model’s ability to learn the behavior of the ultra-high-strength tail. Accordingly, the ≥ 180 MPa subgroup exhibits much larger test-set errors than the better-represented mid-strength ranges, with mean values of RMSE = 29.45 ± 7.41 MPa and MAE = 23.69 ± 6.42 MPa, together with a consistent negative bias of −22.42 ± 7.12 MPa, indicating systematic underprediction of extreme-strength UHPC. These errors are far larger than the aggregate CatBoost performance (RMSE ≈ 4.5 MPa; MAE ≈ 3.6 MPa), showing that the overall metrics are dominated by the densely populated mid-strength regime. Because subgroup sample counts are limited and the within-class variance is relatively narrow, RMSE, MAE, and mean bias are more informative than subgroup R² for this comparison. Therefore, while the proposed model is reliable in the better-represented strength ranges, predictions in the ≥ 180 MPa region should be interpreted with caution, and additional data in the ultra-high-strength regime are needed to improve robustness.

Although the boosting-based models achieved better performance than the ANN in the present experiments, this comparison should be interpreted within the scope of the tested model configurations and tuning protocol. In this study, the ANN was optimized over a limited set of feed-forward architectures, with search ranges of 1–3 hidden layers, 32–128 neurons per layer, ReLU/Tanh activation, batch size 16–64, and maximum epochs 100–300. Therefore, the current results indicate that boosting-based methods were more effective within the explored search space, but they do not establish a general superiority of gradient boosting over all possible ANN architectures for UHPC strength prediction. The lower performance of ANN is consistent with the moderate dataset size and the constrained network architecture adopted to reduce overfitting.

## 4. Model explainability

### 4.1. SHAP-based Analysis

**Fig 6:**
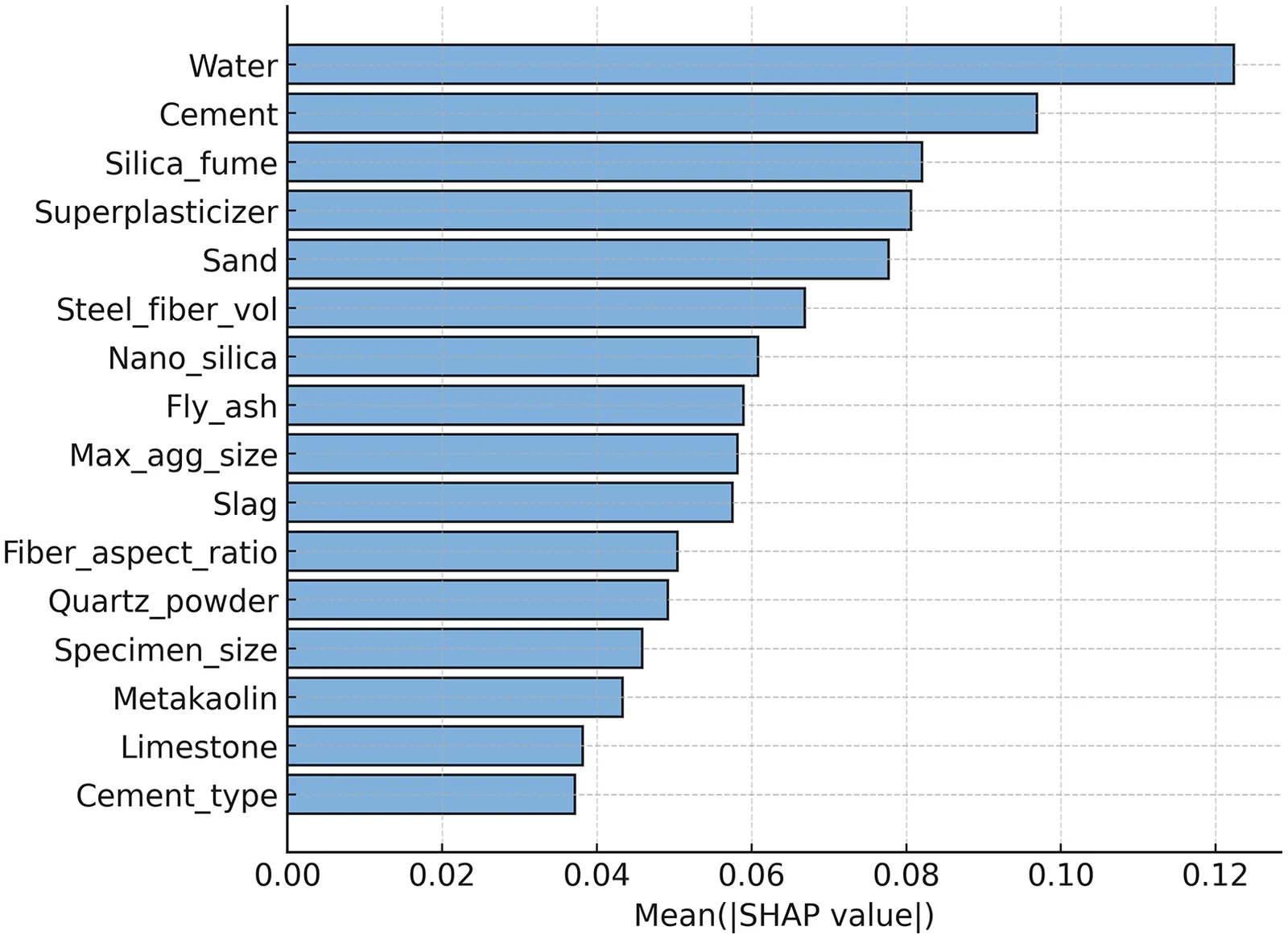
Mean absolute SHAP values.

To interpret the internal behavior of the best-performing model and identify the most influential input parameters affecting the compressive strength of UHPC, SHapley Additive exPlanations (SHAP) analysis was conducted. Fig 6 presents the mean absolute SHAP values of all input features, reflecting their relative importance in the predictive process.

The SHAP results show that water content is the most dominant factor affecting the compressive strength of UHPC, followed by cement, silica fume, superplasticizer and sand content. The remaining factors, including steel fiber volume, nano silica and fly ash, also have insignificant contributions. Parameters such as metakaolin, limestone and cement type have relatively small impacts.

The water variable exhibits the highest mean SHAP value. It highlights its strong negative correlation with compressive strength. A lower water/binder ratio results in denser particle packing. This reduces porosity and increases the cohesiveness of the matrix, all of which increase strength. Conversely, excessive water content results in a more porous microstructure and lower mechanical performance. The strong negative contribution of water-related parameters is consistent with fundamental UHPC principles. A low water-to-binder ratio promotes matrix densification and reduces capillary porosity, leading to improved compressive strength. Excessive water content disrupts particle packing and weakens the interfacial transition zones, which explains the pronounced decrease in predicted strength observed in the SHAP and PDP analyses.

Cement content is ranked as the second most influential factor. Higher cement content generally increases the available hydration products and improves matrix bonding. Both silica fume and superplasticizer have positive SHAP values. This illustrates how they work together to improve the pore structure and workability of UHPC concrete. As a highly reactive pozzolan, silica fume fills micropores and strengthens the interface transition zone (ITZ). Superplasticizer ensures uniform hydration and compactness by optimizing the dispersion of tiny particles. The positive influence of silica fume identified by SHAP and PDP analyses aligns with its well-established role in UHPC. Silica fume enhances particle packing density and contributes to secondary pozzolanic reactions, refining the pore structure and strengthening the cementitious matrix. The observed threshold behavior reflects the balance between matrix densification and potential workability or agglomeration effects at higher contents.

Sand and steel fibers also play an important role. Sand influences the grain structure and distribution. Steel fibers contribute to load transfer and crack filling, improving strength. Although nano-silica and fly ash are of lesser importance for SHAP, they still positively influence strength by enhancing microstructural densification and secondary hydration reactions. Metakaolin, limestone powder, and cement type exhibit relatively small SHAP values. Their influence is less direct or becomes significant only under specific compositional or curing conditions. The moderate but consistent contribution of steel fiber content reflects its indirect role in compressive strength enhancement. While fibers primarily improve tensile and post-cracking behavior, their presence can constrain microcrack propagation under compression and improve load transfer within the matrix, resulting in the observed positive SHAP values.

### 4.2. SHAP waterfall analysis

**Fig 7:**
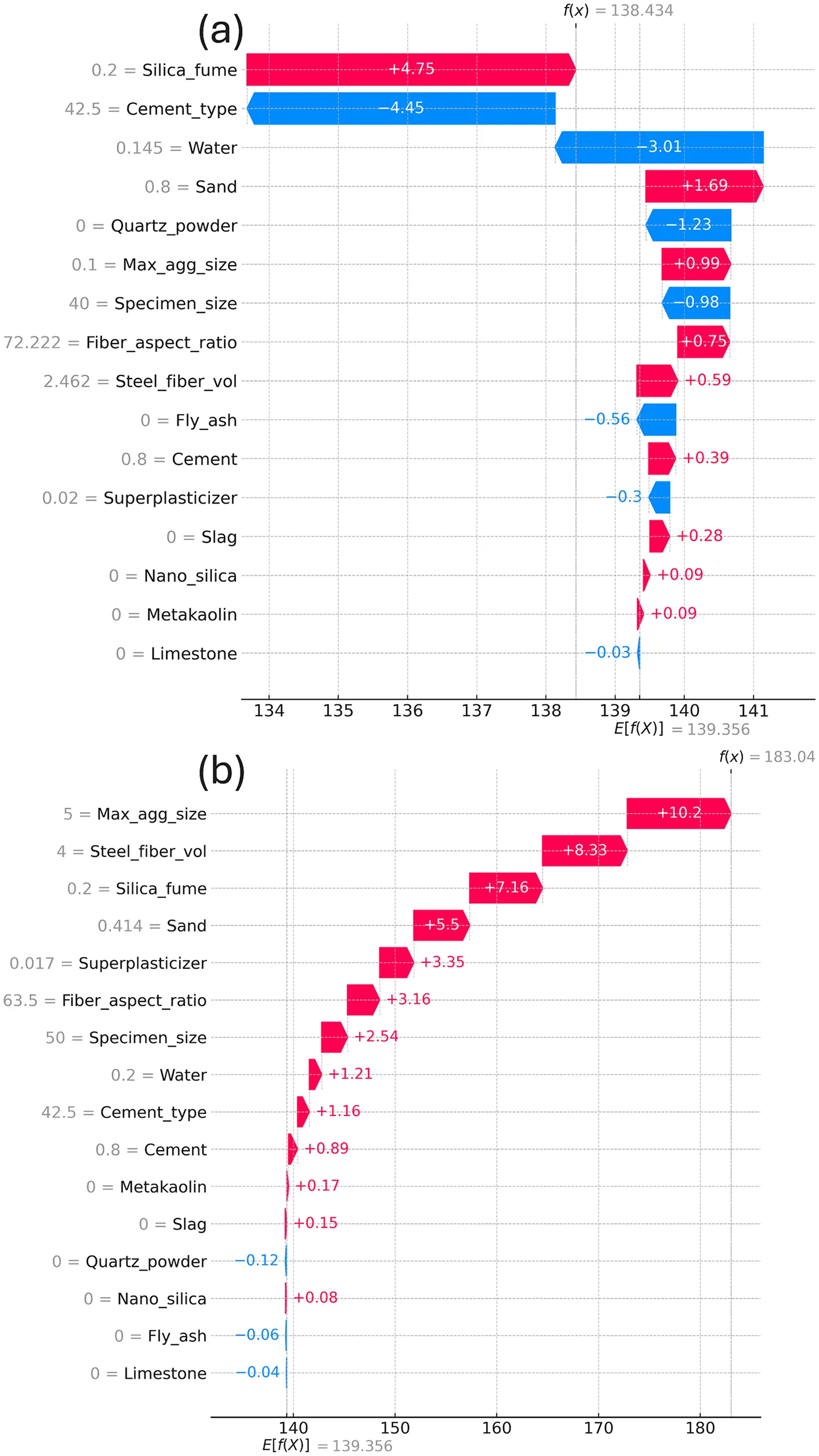
SHAP waterfall: (a) Medium-strength UHPC sample; (b) High-strength UHPC sample.

To complement the global feature importance and dependency results, SHAP waterfall plots were used to analyze the contribution of each input feature to individual sample predictions. Fig 7 provides a local interpretability view, showing how each variable either increases or decreases the predicted compressive strength relative to the model’s baseline mean prediction

In the case of the medium strength UHPC sample (Fig 7a), the baseline model prediction was approximately 139 MPa, and the final prediction for the sample was 138.4 MPa. The waterfall plot illustrates the contribution of both positive and negative characteristics to this result. The most significant positive factors were silica fume (+4.75 MPa) and sand (+1.69 MPa). They helped increase the compressive density and pozzolanic activity, leading to higher predicted strength. Cement type (−4.45 MPa) and water (−3.01 MPa) acted as the main negative influences. This is consistent with the known adverse effects of higher water ratios or lower cement contents. Other variables, such as superplasticizer (+0.28 MPa) and steel fiber volume (+0.59 MPa), provided minor but positive effects, whereas the presence of fly ash (−0.56 MPa) and quartz powder (−1.23 MPa) contributed slightly to strength reduction. This sample demonstrates a well-balanced mix composition, where the beneficial effects of silica fume and sand are offset by the unfavorable influence of higher water content and lower cement grade.

The second case corresponds to a UHPC mix with a predicted compressive strength of 183 MPa (Fig 7b). This compressive strength is much higher than the average of the data set. The base model value is again approximately 139 MPa, implying a net positive contribution of approximately +44 MPa from the input characteristics. The maximum aggregate size (+10.2 MPa), steel fiber volume (+8.33 MPa) and silica fume (+7.16 MPa) are the main contributors. This is followed by sand (+5.5 MPa), superplasticizer (+3.35 MPa) and fiber ratio (+3.16 MPa). These results show that the optimal aggregate gradation, effective fiber reinforcement and well-balanced binder composition contribute significantly to the enhancement of UHPC strength. Small positive contributions were observed from water (+1.21 MPa), cement type (+1.16 MPa) and cement content (+0.89 MPa). The low but controlled water/binder ratio and high reactivity of the binder further improved the performance. Negative effects from quartz powder (−0.12 MPa), fly ash (−0.06 MPa) and limestone (−0.04 MPa) were negligible. These components played a minor role in this optimized mix.

Comparing these two cases highlights the contrasting roles of key mix parameters at different performance levels. For medium-strength UHPC, the type of water and cement dominates the negative side of the prediction. For high-strength UHPC, fiber and aggregate-related parameters lead to positive deviations. The results emphasize the interdependence between binder chemistry, fiber reinforcement, and aggregate structure in achieving superior compressive strength.

The SHAP-based ranking of input variables is consistent with findings reported in previous experimental and data-driven studies on UHPC. In particular, the dominant influence of water-related parameters identified by the model aligns well with extensive experimental evidence showing that a low water-to-binder ratio is essential for achieving high UHPC compressive strength due to enhanced matrix densification and reduced capillary porosity [40–42]. Similarly, the strong positive contribution of silica fume observed in this study corroborates earlier research demonstrating that silica fume improves particle packing density and promotes secondary pozzolanic reactions, leading to a denser microstructure and higher compressive strength [36,43,44]. The moderate contribution of steel fiber content revealed by the SHAP analysis is also consistent with previous studies, which report that fibers primarily enhance tensile and post-cracking behavior, while their effect on compressive strength is indirect and relatively limited [45]. The agreement between the model-derived feature importance and established UHPC material behavior suggests that the machine learning models capture physically meaningful relationships rather than spurious statistical correlations.

SHAP waterfall analysis provides detailed interpretation at the sample level. This allows researchers to understand how each mix component contributes to the final prediction. This capability enhances the transparency of machine learning models and provides practical guidance for optimizing UHPC formulations under different design constraints.

### 4.3. SHAP interaction analysis for second-order effects

**Fig 8:**
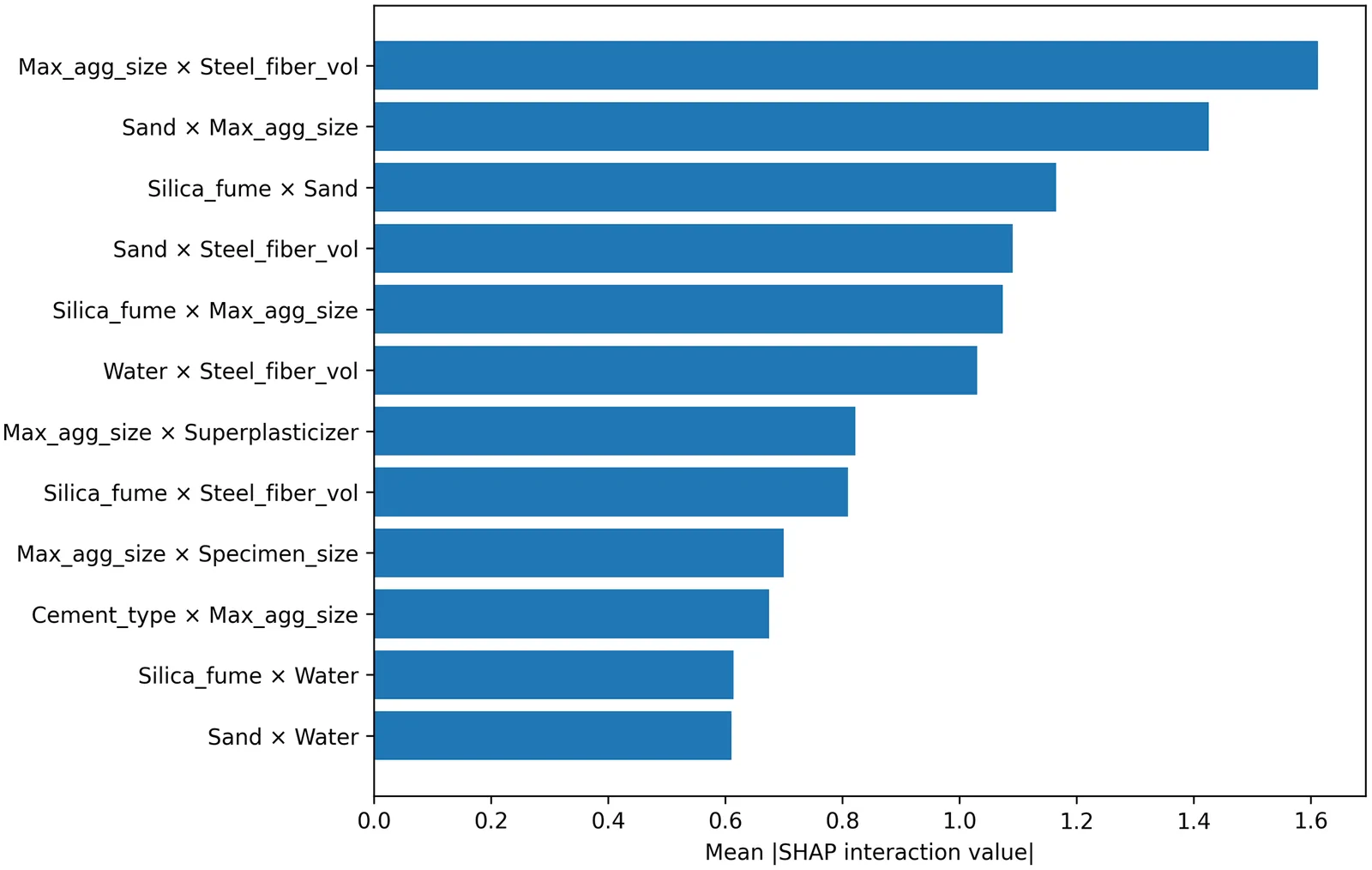
Global ranking of pairwise feature interactions based on mean absolute SHAP interaction values.

To address the limitation of first-order SHAP feature ranking, we further analyzed second-order effects using TreeSHAP interaction values on the hold-out test set (XGBoost model). The global interaction ranking (Fig 8) shows that the strongest pairwise interaction in the present dataset is steel fiber volume × maximum aggregate size (mean |SHAP interaction| = 1.612), while silica fume × water is moderate (rank #11, mean = 0.614) and silica fume × specimen size is weak (rank #47, mean = 0.081).

**Fig 9:**
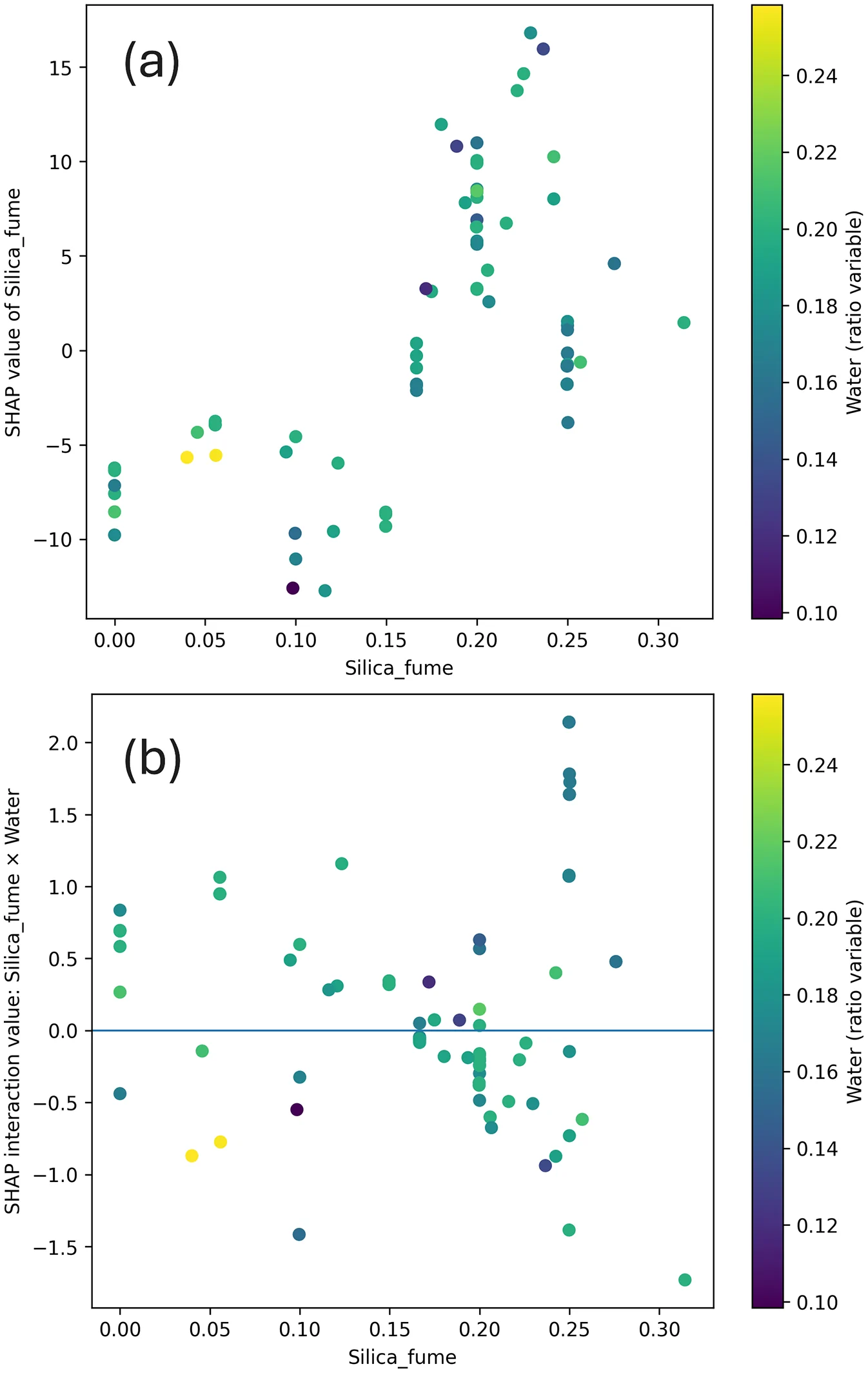
SHAP-based analysis of the silica fume × water interaction: (a) SHAP dependence of silica fume colored by the water-related variable; (b) SHAP interaction values for silica fume × water.

The SHAP dependence/interaction plots (Fig 9) indicate that the effect of silica fume is conditional on the water regime rather than globally monotonic, with a more favorable contribution concentrated in a mid-range silica-fume region.

**Fig 10:**
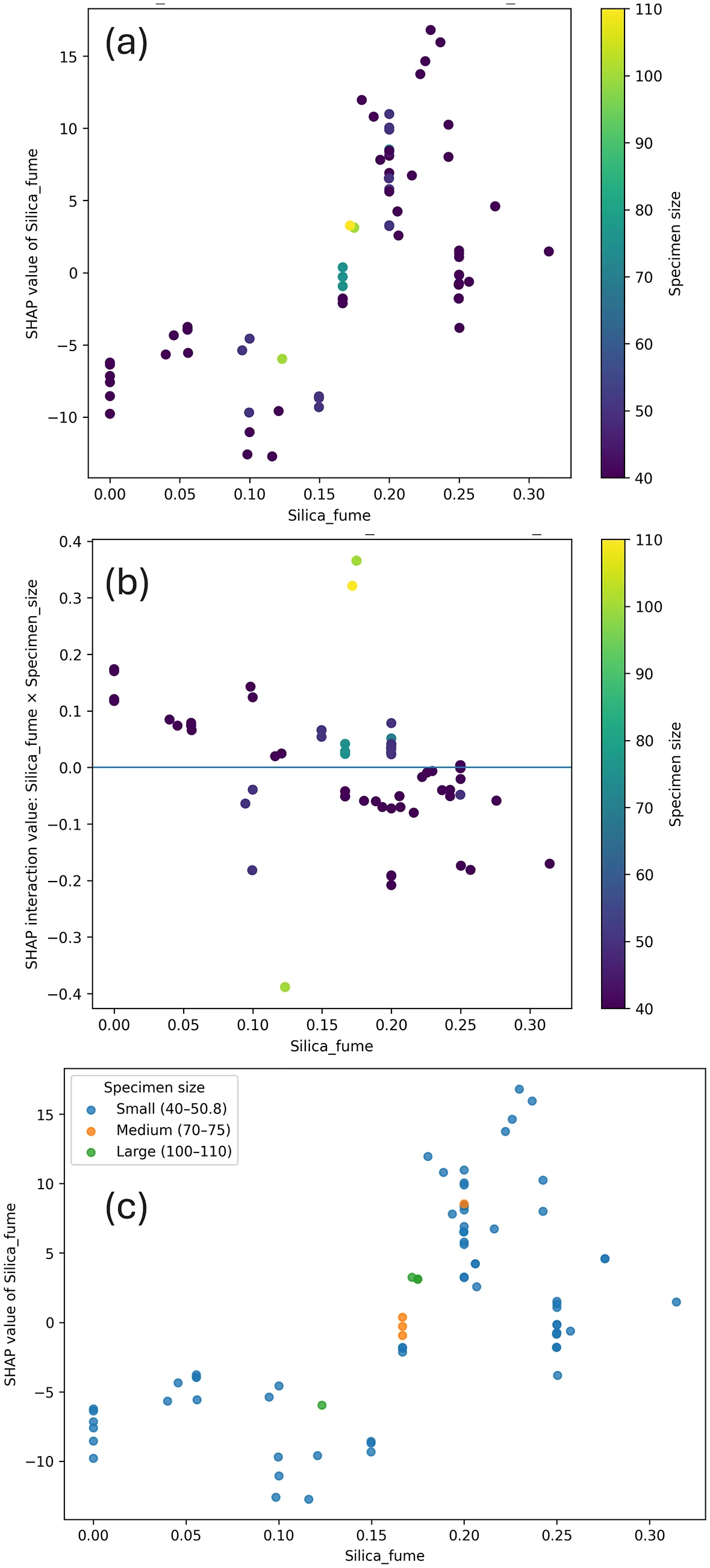
SHAP-based analysis of the silica fume × specimen size relationship: (a) silica-fume dependence plot colored by specimen size; (b) SHAP interaction values for silica fume × specimen size; (c) stratified silica-fume SHAP response by specimen-size groups.

In contrast, the silica fume × specimen size plots (Fig 10) do not provide strong evidence for a robust specimen-size-dependent shift in the apparent silica-fume threshold, and this interpretation is further limited by sparse data in some specimen-size/silica-fume combinations.

**Fig 11:**
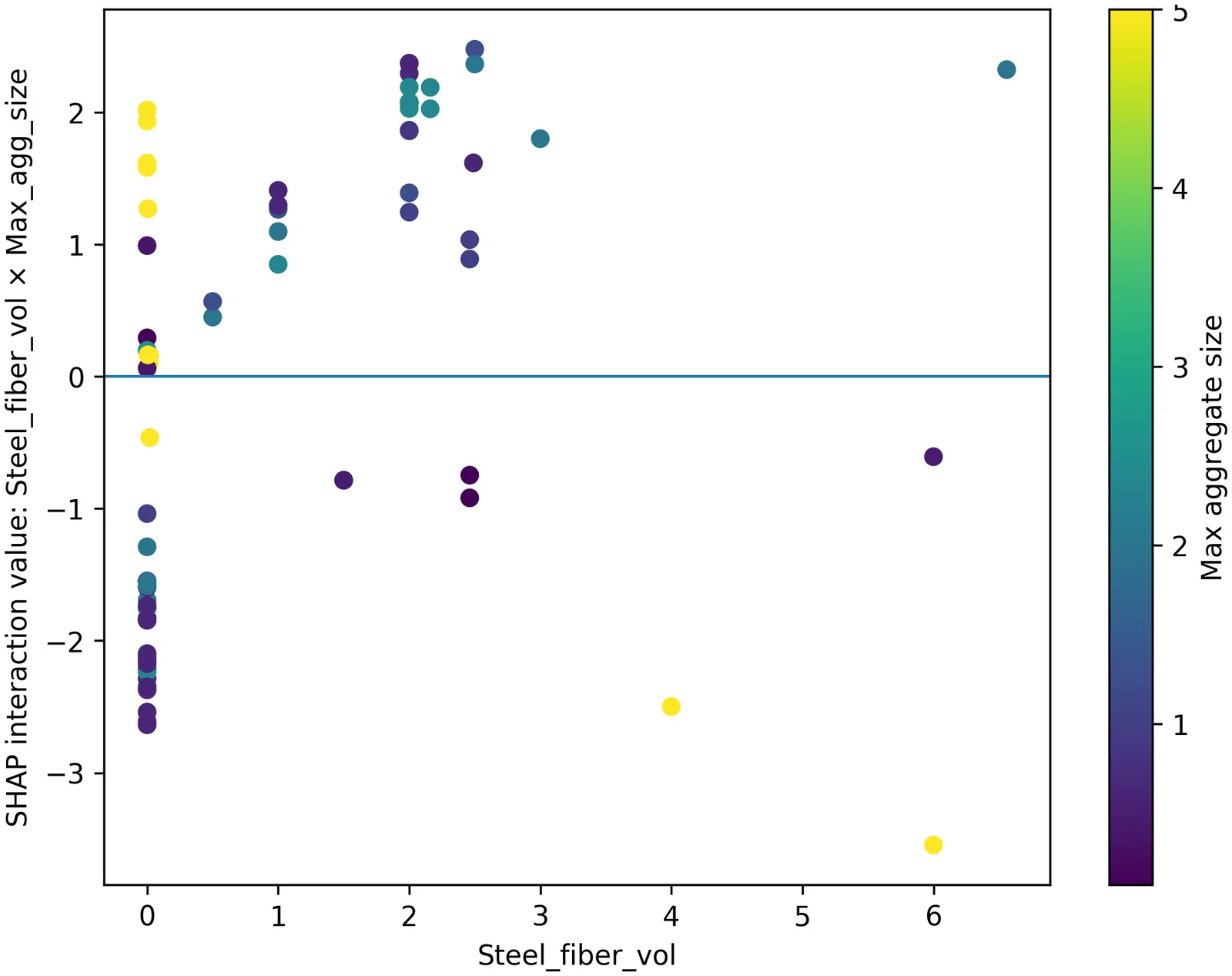
SHAP interaction plot for steel fiber volume × maximum aggregate size.

A key finding is that steel fiber volume × maximum aggregate size emerges as the dominant hidden coupling (Fig 11), suggesting that fiber effectiveness depends on the aggregate-size regime/packing condition. This interaction analysis shifts the interpretation from a simple importance hierarchy to an interaction-aware design space and provides a stronger scientific contribution than feature ranking alone. SHAP interactions are interpreted here as model-learned associations within the observed data domain rather than direct causal mechanisms.

These findings suggest that specimen-size-related effects are secondary in the present model and should be interpreted cautiously. In a literature-compiled dataset, Specimen_size may absorb part of the heterogeneity associated with specimen geometry and test setup, but it should not be viewed as an intrinsic material driver in the same sense as water, cement, or silica fume. This interpretation is consistent with the weak interaction strength observed for silica fume × specimen size and with the broader SHAP/PDP results, which identify water, silica fume, superplasticizer, steel fiber volume, and cement as the dominant predictors of UHPC compressive strength. Accordingly, the primary mechanistic conclusions of this study are drawn from the compositional variables, whereas specimen-size effects are discussed only as a secondary source of experimental heterogeneity.

### 4.4. ICE and PDP

**Fig 12:**
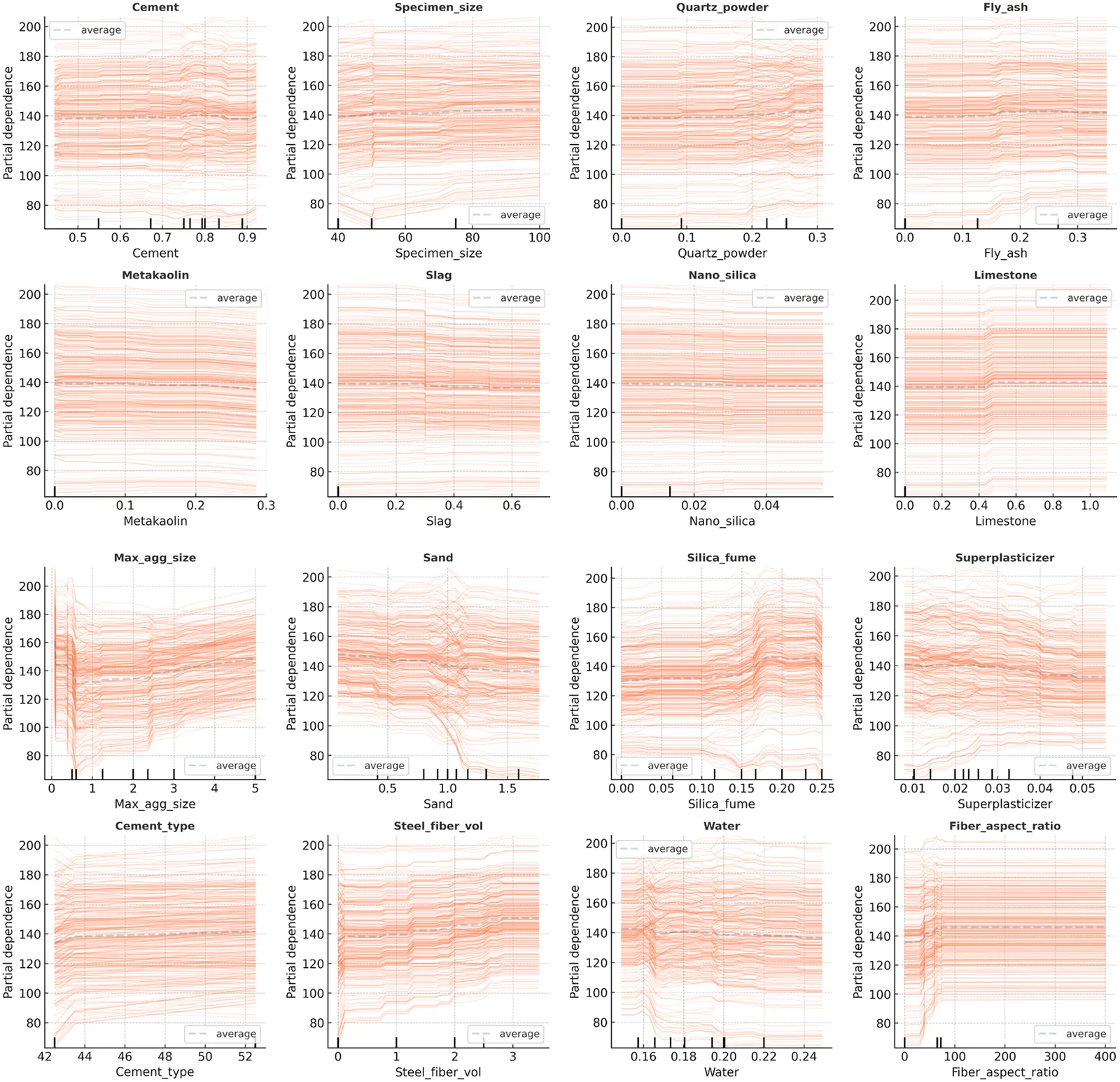
Individual Conditional Expectation (ICE) and Partial Dependence (PDP).

To provide a deeper understanding of how individual input parameters influence the predicted compressive strength of UHPC, Individual Conditional Expectation (ICE) and Partial Dependence Plots (PDP) were generated for all features, as shown in Fig 12. These plots visualize both the global trends (via PDPs) and local sample-level variations (via ICE curves), allowing a detailed interpretation of nonlinear dependencies and potential feature interactions captured by the CatBoost model.

The ICE and PDP plots reveal that the most influential parameters affecting UHPC compressive strength are water, silica fume, superplasticizer, steel fiber volume, and cement, which aligns well with the SHAP-based importance ranking discussed earlier. A strong negative relationship is observed between water content and compressive strength. Both PDP and ICE curves show that increasing the water-to-binder ratio beyond approximately 0.20 results in a sharp decline in predicted strength, due to higher porosity and reduced matrix density. Below this threshold, the strength increases rapidly, confirming that a low water ratio enhances packing efficiency and hydration compactness. The plots demonstrate a nonlinear positive correlation between silica fume content and compressive strength. Strength increases significantly up to about 0.20 (by mass ratio), beyond which the curve flattens, indicating an optimal dosage range. This reflects the dual role of silica fume in improving particle packing and contributing to secondary hydration reactions that refine the UHPC microstructure. A clear positive effect of superplasticizer content is visible at moderate dosages (~0.02–0.04). The predicted strength rises due to improved workability and uniform particle dispersion. However, the curve tends to plateau or slightly fluctuate at higher dosages, suggesting that excessive admixture may lead to segregation or delayed setting. Both parameters exhibit a monotonic positive influence on compressive strength, particularly at low-to-moderate fiber volumes (<2%). The curves reveal that fibers enhance strength by bridging microcracks and redistributing stress, though the marginal gain diminishes at higher contents. The fiber aspect ratio further amplifies this effect up to around 200, after which the improvement becomes less pronounced, likely due to mixing and workability limitations. The plots indicate that increasing cement content enhances strength up to an optimum range (~0.8 by normalized ratio). Different cement grades (represented by cement type) also influence strength predictions, with higher strength cements producing higher PDP values, consistent with the material’s intrinsic hydration capacity.

The combined ICE and PDP results provide a holistic view of the model’s behavior, capturing both the average global dependencies and individual-level nonlinear variations across the dataset. The findings confirm that UHPC compressive strength is controlled by a complex interplay of binder composition, water-to-binder ratio, and fiber reinforcement characteristics.

The tight clustering of ICE curves for most variables indicates model stability and low sensitivity to noise, while the moderate spread observed for features such as superplasticizer and fiber volume highlights interactive effects with other mix components.

The nonlinear trends revealed by the PDP and ICE analyses further support well-established UHPC principles reported in the literature. The threshold behavior observed for silica fume content is consistent with experimental studies indicating that excessive silica fume may lead to particle agglomeration or reduced workability, which can limit further strength development [36,46–48]. These consistencies between the model interpretations and prior experimental observations further enhance the credibility and dependability of the proposed machine learning framework.

**Fig 13:**
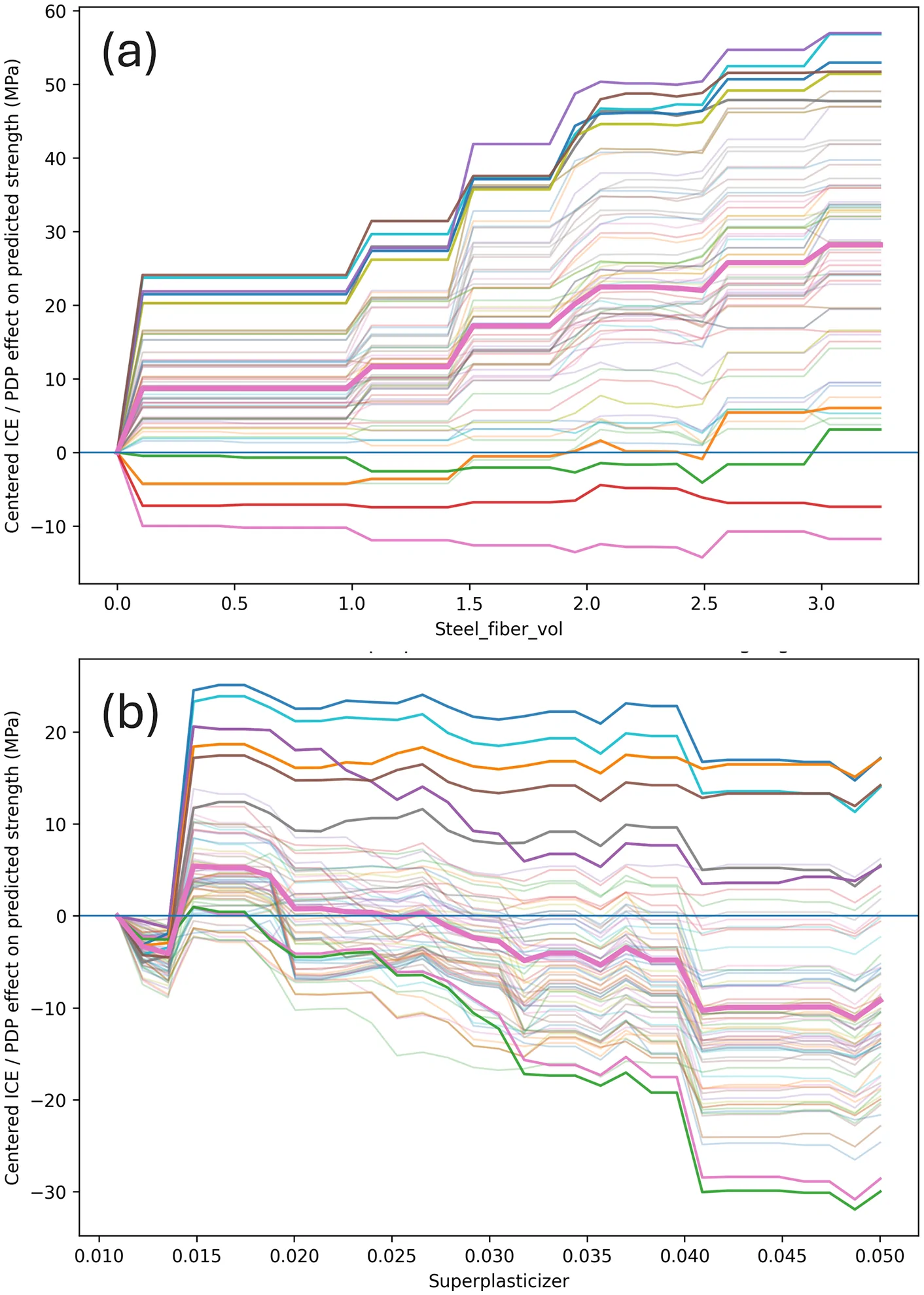
Centered ICE (c-ICE) curves and PDP trends with outlier ICE curves highlighted for variables showing substantial sample-level heterogeneity: (a) Superplasticizer and (b) Steel fiber volume.

To clarify the non-redundant roles of PDP and ICE, we further examined the centered ICE (c-ICE) spread for Superplasticizer and Steel fiber volume (Fig 13), with the outlier ICE curves highlighted. While the PDP captures the average marginal trend, the visible spread of c-ICE curves indicates substantial sample-level heterogeneity, i.e., the effect of these variables is not uniform across mixtures. For Superplasticizer, the outlier ICE curves show both strongly positive and strongly negative local responses relative to the average trend, confirming that superplasticizer dosage has a regime-dependent effect rather than a single monotonic contribution. Profiling the samples associated with these outlier curves showed that they are more frequently associated with higher silica-fume content and smaller maximum aggregate size (rather than a uniformly low-water condition), suggesting that the marginal benefit of superplasticizer is amplified in mixtures with higher powder demand and finer aggregate skeletons. For Steel fiber volume, the c-ICE spread is also pronounced, with some mixtures showing large gains and others showing weak/near-flat responses as fiber volume increases. The outlier fiber-response curves are associated with a distinct aggregate-size regime (higher maximum aggregate size in the present dataset), consistent with the interaction-aware interpretation that fiber effectiveness depends on the surrounding packing/skeleton condition. The c-ICE analysis complements the PDP results by revealing mixture-specific responses that are not visible from the average marginal trend. The outlier ICE curves reveal regime-dependent design tendencies that can be used as preliminary mixture-design guidance. These trends are interpreted as model-learned associations within the observed data domain rather than causal design rules.

The specific outlier samples (IDs and compositions) were traced and checked during the ICE profiling step, representative profiles are summarized in the text and can be provided in the supplementary material if required.

The use of XAI techniques is important because high prediction accuracy alone is not sufficient for engineering decision-making in UHPC mixture design [49]. In practical applications, engineers need to understand why a model predicts a higher or lower compressive strength and how specific mixture variables contribute to this prediction. SHAP, PDP, and ICE analyses provide this additional layer of interpretation by identifying influential variables, revealing nonlinear effects, and showing whether the influence of each variable is consistent across different mixtures. For UHPC, this interpretability is particularly useful because compressive strength is governed by coupled effects among water content, binder composition, silica fume, superplasticizer dosage, aggregate characteristics, fiber parameters, and specimen-related factors. By linking these variables to predicted strength, XAI can help identify favorable mixture-design trends, detect unrealistic model behavior, and support more transparent decision-making. Thus, the role of XAI in this study extends beyond post-processing visualization. It provides a bridge between machine learning predictions and physically meaningful UHPC mix-design interpretation.

## 5. Conclusions

This study developed a comparative and interpretable machine-learning framework for predicting the 28-day compressive strength of UHPC using a multi-source database comprising 379 mixtures and 16 input variables. The main conclusions are summarized as follows:

(1) CatBoost achieved the best overall performance, with R^2^ > 0.90, RMSE of approximately 4.5 MPa, and MAE of approximately 3.6 MPa. LightGBM and XGBoost also showed competitive performance.
(2) Prediction reliability varied across the strength range. The model performed well in the well-represented ranges but showed larger errors and systematic underprediction for mixtures with compressive strength ≥180 MPa.
(3) Water, cement, silica fume, superplasticizer, sand, and fiber-related parameters were identified as the most influential variables. Their effects were generally consistent with established UHPC material behavior.
(4) SHAP, PDP, ICE, and interaction analyses revealed nonlinear and mixture-dependent relationships among the input variables. The proposed framework can therefore support preliminary and data-informed UHPC mixture assessment, but it should not replace experimental validation.

Limitations of the study: The database was moderate in size and imbalanced in the ultra-high-strength range. Its multi-source nature also introduced heterogeneity in materials, curing conditions, specimen geometry, and testing procedures. Several relevant variables were unavailable, and independent external validation was not conducted. Moreover, the XAI results represent statistical associations rather than causal design rules.

Future research directions: Future studies should develop larger and more standardized databases, particularly for mixtures exceeding 180 MPa, incorporate additional curing and testing information, and evaluate model generalizability using source-wise splitting and independent experimental datasets. Uncertainty quantification and physically constrained machine-learning methods should also be investigated.

## Supporting information

S1 File UHPC_data infor. (XLSX)
